# Exposure to workplace violence and threats and risk of depression: a prospective study

**DOI:** 10.5271/sjweh.3976

**Published:** 2021-10-31

**Authors:** Laura A Rudkjoebing, Åse Marie Hansen, Reiner Rugulies, Henrik Kolstad, Jens Peter Bonde

**Affiliations:** 1 Department of Occupational and Environmental Medicine, Bispebjerg University Hospital, Copenhagen, Denmark; 2 Department of Public Health, University of Copenhagen, Copenhagen, Denmark; 3 National Research Centre for the Working Environment, Copenhagen, Denmark; 4 Department of Psychology, University of Copenhagen, Copenhagen, Denmark; 5 Department of Occupational Medicine, Aarhus University Hospital, Aarhus, Denmark

**Keywords:** anti-depressive medication, bullying, longitudinal study, SCAN interview, workplace harassment

## Abstract

**Objectives:**

Several studies have examined the health consequences of workplace threats and violence, however, due to methodological issues the epidemiological evidence is limited. The purpose of this study was to examine the prospective association between self-labelled exposure to work-related threats and violence and the risk of depression, measured by a standardized psychiatric interview and new prescriptions of anti-depressive medication.

**Methods:**

Employees were recruited from the Danish PRISME cohort established in 2007 where 4489 Danish public employees answered a postal questionnaire with follow-ups in 2009 and 2011. In all three waves, depression was diagnosed by clinical interviews with the Schedules for Clinical Assessment in Neuropsychiatry (SCAN). In addition, we ascertained prescription of anti-depressive medication from a national register. Using logistic regression and Cox proportional hazard models, we estimated the association between exposure to threats and violence at baseline and SCAN depression and prescription of anti-depressive medication during two years of follow-up.

**Results:**

Self-labelled exposure to work-related threats and violence was associated with a risk of SCAN diagnosed depression two years later, odds ratios (OR) 2.20 [95% confidence interval (CI) 1.13–4.28] and OR 2.11 (95% CI 1.05–4.24), respectively, with indication of a dose–response. Self-labelled exposure to work-related threats and violence was associated with prescription of anti-depressive medication in a two-year period, hazard ratios (HR) 2.55 (95% CI 1.47–4.40) and HR 1.47 (95% CI 0.70–3.06), respectively.

**Conclusion:**

Exposure to work-related threats or violence is associated with an increased risk of depression two years later, measured with a psychiatric interview and register data on prescribed antidepressants.

Being threatened with or exposed to physical violence in the workplace is a serious occupational hazard, and work-related threats and violence have received increased attention in recent years. The prevalence of workplace threats and violence varies considerably according to occupational setting, definitions and measurement methods (1). Furthermore, there is a difference in how countries require employers to assess and prevent violence in the workplace (2). The healthcare sector has been the focus of most studies in this area. Among nurses in eight European countries, 22% reported frequent violent episodes from patients and relatives (3), and a recent meta-analysis similarly reported a one-year prevalence of 19% for physical violence against healthcare professionals (4).

Several negative work functioning consequences of exposure to work-related violence have been highlighted, such as malpractice, low quality of care, absenteeism and high turnover (5). In addition, acute and long-term psychological and emotional consequences for individual well-being have been reported (1). However, in longitudinal studies with long follow-up reverse causation is an issue: workplace violence may cause occupational stress, and subsequently the distressed employee may be more exposed to violence than other workers (6, 7).

In a recent review we assessed the association between workplace violence and poor mental health, especially the risk of developing depressive disorder (8). Most studies addressing major depression had important methodological limitations. Many studies were cross-sectional and depression ascertainment relied mostly on self-reported symptoms (8). Only one study explicitly addressed the risk of medically diagnosed depressive disorder (although together with other mood disorders) (9). Two cohort studies used the prescription of anti-depressive medication as a proxy for depressive disorder (10, 11). The results from these three studies suggested an increased risk of depression after exposure to violence or threats of violence at work. Recently a Danish job exposure matrix study on violence at work and depression reported that being employed in jobs with a high likelihood of work-related violence (eg, police offices, prison guards, teachers and personal care workers) was associated with a modest risk of developing depression, measured by hospital treatment in registers (12). The study was based on an inception cohort including a large sample with more than 950 000 Danish employees followed for 6.9 million person-years. For women the association was found across industries while for men it was only found for certain industries (12). The findings support the notion that the association between work-related violence and depression may be causal, however, measuring work-related violence using a JEM may have resulted in misclassifications of exposure.

Measuring clinical depression in epidemiological studies is challenging as different methods have both advantages and disadvantages. The gold standard measure is a psychiatric interview (usually either a structured clinical interview or a clinical diagnostic interview), conducted by a trained interviewer, allowing diagnoses in accordance with the International Classification of Diseases and Related Health Problems (ICD) by the World Health Organization or the Diagnostic and Statistical Manual of Mental Disorders (DSM) by the American Psychiatric Association (13). However, it is a major limitation of psychiatric interviews that they ascertain depressive disorders only at a single point of time during follow-up. Consequently, individuals who developed a depressive disorder shortly after baseline but are in remission at the time of the follow-up interview may be misclassified as non-cases. Further, individuals who do not show up at the follow-up interview are lost to the study. Misclassification of cases and loss to follow-up can be avoided when study participants are continuously monitored in national health registers that provide the exact date when an individual, for example, purchases anti-depressive medications. However, the major disadvantages of prescription databases are that they only identify those cases of depressive disorders where the individual contacted the healthcare system and was subsequently diagnosed, causing selection bias related to healthcare seeking behavior (14). Further, there is a problem concerning the specificity of measuring only depression, as anti-depressive medication is also used to treat conditions such as anxiety disorders and post-traumatic stress disorder (15, 16).

The aim of this study was to examine the prospective association between exposure to workplace violence or threats of violence and depression, while using both a standardized psychiatric interview and a national prescription drug register to measure onset of depression. To the best of our knowledge, no previous studies have examined the association between workplace violence and depression using a standardized psychiatric interview. Further, only one previous study on work-related violence has combined different types of depression assessment (the use of psychotropic medications and health claims for treatment of depression) (10) as we did by combining a psychiatric interview with register data on prescribed antidepressants. We reasoned that if we found an association between threats and violence and depression in both measures of depression, we would feel more confident that the association indicates a causal relation than if we were to find an association for only one measure of depression.

## Methods

### Study design and population

**Figure 1:**
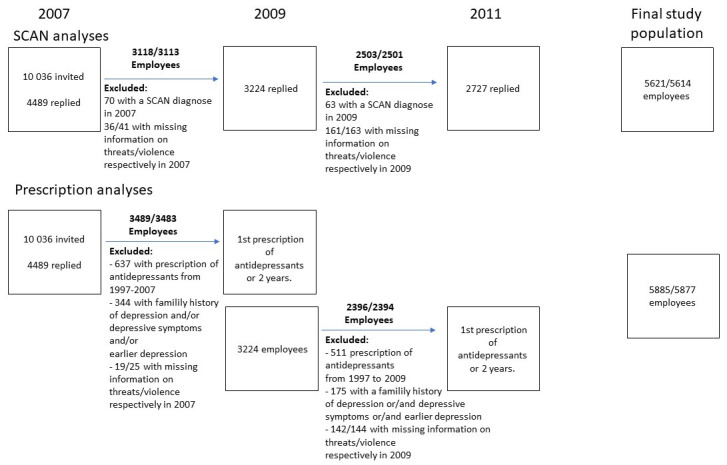
The final study population.

This study examines the longitudinal association between exposure to violence or threats of violence at work and depression. In the first part of the study, depression was diagnosed using a standardized psychiatric interview, the Schedules for Clinical Assessment in Neuropsychiatry (SCAN) study, and we assessed the risk of depression two years after self-reported exposure to violence or threats of violence. In the second part – the prescription study – we examined the risk of prescription of antidepressants after exposure to work-related violence or threats in a period of two years. Data was retrieved from the Danish PRISME study (17, 18). The structure of the study population is shown in figure 1. In the first wave of the study in 2007, 10 036 employees were recruited within public service workplaces in the county and municipality of Aarhus, Denmark and a total of 4489 employees (45%) participated by completing a postal questionnaire concerning working conditions and health. These 4489 participants were then sent a similar questionnaire in 2009 with a response rate of 72% (3224 employees) and again in 2011 with a response rate of 73% (3278 employees). In the SCAN study, we excluded all individuals with an ICD-10 depression diagnosis at baseline (N=100 in 2007 and N=83 in 2009), and only included participants who responded to two subsequent surveys, N=5803. Missing answers on exposure to threats or violence were 3% in both cases, resulting in a final study population of 5621 employees in the analyses regarding threats and 5614 employees in analyses of violence.

In the prescription study, we excluded employees with previous prescriptions of antidepressants (from 1997 to baseline), N=890 in 2007 and N=717 in 2009, and employees with previous self-reported episodes of depression (N=290), a family history of depression (N=1585) and depressive symptoms at baseline (N=272), based on the six-item subscale SCL-DEP6 for depression in the Mental Disorder Questionnaire (CMDQ), cases with a sum-score ≥3 in ≥2 questions were defined as cases of depressive symptoms (19). This resulted in a population of 4678 employees but, because of missing exposure information of 3%, we ended up with 4524 and 4519 employees in the analyses regarding threats and violence, respectively.

### Measures of violence and threats of violence

The participants were asked if during the past 12 months they had been exposed to five different acts: 1 (verbal or written threats), 2 (threatening behavior), 3 (push, minor blow, kicks, bites), 4 (violence that threatens your health) and 5 (life-threatening violence). The first two questions measured threats and the last three violence. Each question had five response categories, which we combined into three to obtain strata with reasonable numbers, namely ‘never’ (comprising the category ‘no’), ‘occasionally’ (comprising ‘yes, one time’ and ‘yes, two-five times’), and ‘frequently’ (comprising the response categories ‘yes, six-ten times’ and ‘yes, more than ten times’). Analyses were also performed using continuous-scale exposure information.

Furthermore, we calculated an index combining the frequency and severity of the threatening or violent acts. For threats, the severity score was 1 (verbal or written threats) and 2 (threatening behavior) and for violence the severity score was 1 (push, minor blow, kicks, bites), 2 (violence that threatens your health) and 3 (life-threatening violence), with higher values indicating more severe acts. The frequency was scored as either 0 (never), 1 (once), 2 (2–5 times), 3 (6–10 times) or 4 (>10 times). The severity-frequency index was calculated by multiplying each severity score by the frequency score and then adding them together separately for threats and violence, resulting in a value of 0–12 for threats and a value of 0–24 for violence. The following example illustrates this index: an employee reporting exposure to verbal or written threats (severity score 1) 6–10 times (frequency score 3) and exposure to threatening behavior (severity score 2) once (frequency score 1) will have a severity-frequency index of 5 for threats (1 × 3 + 2 × 1). The distribution of the index variable is strongly skewed to the left reflecting that frequent exposure to more severe violence is infrequent (supplementary material www.sjweh.fi/article/3976, figures A and B). The index is reported continuously. Exploratory analyses bases eg, on tertiles of the index variable distribution was not possible due to the strongly skewed distribution, but division into equidistant groups based on the value of the index variable indicates, that risk is increasing monotonously by increasing index value (data not shown). However, the analyses based upon grouping of the index variable does not add to the results already shown by the analyses based upon the frequency of threats/violence.

### Measures of diagnosis of depression using the SCAN interview

Depression was diagnosed according to the International Classification of Diseases, Tenth Revision, Diagnostic Criteria for Research (ICD-10-DCR) (20) by applying the SCAN interview, version 2.1, part I. Section 3 (worrying and tension), section 4 (panic anxiety and phobias) and sections 6–8 (depression) were used (21). Each interview took about an hour and focused on the recent 3–5 months. It was computer aided and semi-structured and was conducted by psychology and medical students trained to manage these interviews (22).

Participants in the SCAN interview were selected by four screening criteria, slightly different in the three rounds, and described in detail by Kolstad et al (18) and Gullander et al (22).

### Measures of diagnosis of depression by prescription of antidepressant medication

Following the World Health Organization-developed Anatomical Therapeutic Chemical (ATC) classification system (23), antidepressants were identified with the code N06a. Baseline was set the day the participants answered the questionnaire. The participants were linked to a national registry of prescription medication purchases to detect incident use of anti-depressant medication over a 2-year follow-up period as this was the period between the questionnaires.

### Statistical analysis

All analyses were undertaken on pseudo-anonymized data on a remote platform at Statistics Denmark using SAS 9.4 software (SAS Institute, Cary, NC, USA).

Crude and adjusted odds ratios (OR) and 95% confidence intervals (CI) for the risk of newly onset SCAN depression according to self-reported threats or violence at baseline were calculated using logistic regression. The follow-up period from baseline to ascertainment of outcome was approximately two years and each participant could contribute to ≤2 follow-up periods. The proc Genmod repeated measures procedure accounted for the lack of independence of the ≤2 follow-up periods in the same individual.

Cox proportional hazards models with days as the underlying timescale were used to calculate hazards ratios (HR) and 95% CI for the prospective association between exposure to workplace violence or threats and the risk of prescription of antidepressants. Employees were followed from the date they completed the questionnaire until the first prescription of anti-depressive medication event or the end of follow-up (two years), whichever came first. We performed traditional analyses with threats and violence measured at baseline and first prescription of antidepressant during follow-up (2007–2009 and 2009–2011). Cox regressions were performed applying the PHREG procedure.

The proportional hazards assumption was tested using the assess proportionality statement for all the variables in the final model and the assumption was not violated by any of the variables.

All the analyses were performed with exposure divided into three categories (never, occasionally and frequently, described above) and an exposure severity-frequency index, explained above.

In the final analyses, we included confounders measured in the baseline questionnaire: gender, age (19–29, 30–39, 40–49, 50–59 and >59 years), education beyond primary or high school (<3, 3–4 and >4 years), weekly alcohol consumption (≤7 units, >7 units), smoking (never, former smoker, smoker), neuroticism (0–2, 3–6 on the neuroticism scale of the Eysenck Personality Questionnaire Revised-Abbreviated version), and traumatic life events during the last six months (measured with nine questions on eg, serious illness or injury, assault, death of a relative or friend, marital problems or serious illness or assault of a close relative (24), with a positive answer to a minimum of one question being scored as yes, otherwise scoring as no). In the logistic regression analyses we also adjusted for previous episodes of depression (yes/no), family history of depression (yes/no) and depressive symptoms [based on the six-item subscale SCL-DEP6 for depression in the Mental Disorder Questionnaire (CMDQ) (19)]. In the prescription study, these participants were excluded but due to a smaller sample and fewer cases, we would have lost too much power in the SCAN study. However, we conducted separate analyses, which excluded employees with previous episodes of depression.

We tested for interaction between gender and violence and threats, between participants reporting earlier depression and violence/threats and between occupational group (nurses versus the rest) and violence/threats, all of which were statistically non-significant.

## Results

In the first round (2007), 4489 employees participated (45% of all invited). The prevalence of threats was 37.4% while the prevalence of violence was 18.4%. The baseline prevalence of SCAN depression was 2.2% and 6.8% had a prescription of anti-depressive medication in the year before baseline. The most common professions were nurses (28%), social work and counselling professionals (17%) and teachers (8%).

Table 1 presents baseline characteristics of employees who answered the questionnaire both in 2007 and 2009, the first follow-up of the SCAN study, in relation to violence. The exposure to violence was the same among females and males. Employees reporting frequent exposure to violence were younger, less educated, had a greater tendency to be smokers and reported more traumatic life events during the last six months than employees not exposed to violence. An overlap was seen between threats and violence and employees reporting threats also reported more frequent exposure to violence than employees who did not report threats.

The results of the SCAN analyses are presented in table 2. Employees reporting frequent exposure to threats had an increased risk of depression two years later (OR 2.20, 95% CI 1.13–4.28), with a dose–response and the continuous severity-frequency index also showed an increased risk (OR 1.09, 95% CI 1.02–1.16 per 1-unit increase). Frequent exposure to violence at work also increased the risk of depression two years later, OR 2.11 (95% CI 1.05–4.24) with a dose–response and a significant OR on the continuous scale. The continuous severity-frequency index also showed and increased risk of depression OR 1.12 per 1-unit increase (95% CI 1.02–1.22 per 1-unit increase).

**Table 1:** Baseline characteristics of participants in the first round of the study (2007-2009) according to the exposure to violence at baseline.

	N total=3183		Exposure to violence					

			Never		Occasionally		Frequently	
			
	N	%	N	%	N	%	N	%
Gender								
Female	2501	79	2050	78	316	81	135	78
Male	682	21	570	22	75	19	37	22
Age groups (years)								
19–29	250	8	177	7	51	13	22	13
30–39	720	23	554	21	106	27	60	35
40–49	967	30	785	30	130	33	52	30
50–59	1091	34	959	37	96	25	36	21
>59	155	5	145	5	8	2	2	1
Higher education (years)								
<3	560	18	481	18	49	13	30	18
3–4	2231	70	1768	68	325	83	138	80
>4	385	12	365	14	16	4	4	2
Smoking								
Never	1517	48	1268	49	166	42	36	21
Former	1152	36	962	37	139	36	51	30
Smoker	495	16	374	14	85	22	83	49
Alcohol (units/week)								
≤7	2387	76	1948	75	307	79	132	80
>7	766	24	651	25	81	21	34	20
Traumatic life events								
No	2047	64	1713	65	236	60	98	57
Yes	1136	36	907	35	155	40	74	43
Family depression								
No	2274	73	1891	73	265	69	118	70
Yes	856	27	686	27	119	31	51	30
Earlier depression								
No	2678	86	2214	87	321	84	143	85
Yes	421	14	335	13	61	16	25	15
Depressive symptoms								
No	2973	94	2458	94	357	92	158	92
Yes	203	6	158	6	31	8	14	8
Depression (ICD-10)								
No	3113	98	2565	98	380	97	168	98
Yes	70	2	55	2	11	3	4	2
Neurotic								
No	2661	84	2202	84	319	82	140	81
Yes	522	16	418	16	72	18	32	19
Threats								
Never	2029	64	1903	73	92	24	34	20
Occasionally	911	29	652	25	232	59	27	16
Frequently	240	7	62	2	67	17	111	64

**Table 2:** Results from the logistic regression analysis, showing the risk of newly-onset depression (2007–2009 and 2009–2011) by self-reported threats of violence and violence in 2007 and 2009, respectively. [OR=odds ratio; CI=confidence interval; SCAN=Schedules for Clinical Assessment in Neuropsychiatry.]

	Number of observations	New cases of depression SCAN	%	OR_crude_	95% CI	OR_adj_^a^	95% CI
Threats							
Never	3737	56	1.5	1		1	
Occasionally	1501	27	1.8	1.20	0.76–1.91	1.06	0.64–1.72
Frequently	383	13	3.4	2.30	1.25–4.25	2.20	1.13–4.28
Continuous ^b^				1.42	1.05–1.93	1.35	0.96–1.89
Severity-frequency index ^c^				1.10	1.04–1.16	1.09	1.02–1.16
Violence							
Never	4676	69	1.5	1		1	
Occasionally	663	16	2.4	1.65	0.95–2.86	1.19	0.63–2.24
Frequently	275	10	3.6	2.52	1.29–4.92	2.11	1.05–4.24
Continuous ^b^				1.60	1.18–2.17	1.45	1.04–2.03
Severity-frequency index ^c^				1.13	1.06–1.21	1.12	1.02–1.22

a Adjusted for age, gender, earlier depression, depressive symptoms, family history of depression, higher education, alcohol, smoking, traumatic life events and neuroticism.

b Increase in OR by 1 on a scale ranging from 0-3.

c Increase in OR by 1 on the severity frequency scale (ranging from 0–12 for threats and 0–24 for violence).

The results from the analyses excluding employees with previous episodes of depression are reported in supplementary table B. Point estimates were similar to the estimates in the main analyses (except for occasional threats where the OR was 0.71 compared to 1.04 in the main analysis, although not significant in any of the cases), while CI were wider, indicating lower precision.

Table 3 presents the results from the prescription study. Frequent exposure to threats at work was associated with a higher risk of purchasing prescribed antidepressants within a two-year period (HR 2.55, 95% CI 1.47–4.40). There was an indication of a dose–response association with a continuous scale score of HR 1.48 (95% CI 1.13–1.94) and the severity-frequency index of HR 1.09 (95% CI 1.03–1.15 per 1-unit increase). Regarding violence, we again found a dose–response; however, the adjusted results did not reach the significance level, except for the continuous severity-frequency index (HR 1.09, 95% CI 1.01–1.18 per 1-unit increase).

**Table 3:** Results from the Cox regression analysis, showing the risk of prescription of antidepressants (2007–2009 and 2009–2011) by self-reported threats of violence and violence in 2007 and 2009, respectively. [HR=hazard ratio; CI=confidence interval.]

	Number of observations	New cases of prescription of antidepressant	%	Person-years	Cases per 10 000 person-years	HR_crude_	95% CI	HR_adj_^a^	95% CI
Threats									
Never	3928	69	1.8	7727	89				
Occasionally	1563	31	2.0	3071	101	1.12	0.73–1.71	1.17	0.76–1.82
Frequently	394	17	4.3	763	223	2.47	1.45–4.20	2.55	1.47–4.40
Continuous ^b^						1.44	1.11–1.87	1.48	1.13–1.94
Severity-frequency index ^c^						1.09	1.03–1.14	1.09	1.03–1.15
Violence									
Never	4859	89	1.8	9554	93				
Occasionally	729	20	2.7	1423	141	1.50	0.92–2.44	1.47	0.90–2.42
Frequently	289	8	2.8	560	143	1.51	0.73–3.11	1.47	0.70–3.06
Continuous^b^						1.31	0.97–1.76	1.29	0.94–1.75
Severity-frequency index ^c^						1.10	1.02–1.18	1.09	1.01–1.18

a Adjusted for age, gender, higher education, alcohol, smoking traumatic life events and neuroticism.

b Increase in HR by 1 on a scale ranging from 0–3.

c Increase in HR by 1 on the severity frequency scale (ranging from 0–12 for threats and 0–24 for violence).

## Discussion

We observed a prospective association between self-labelled exposure to frequent violence and threats of violence at the workplace and the occurrence of depression two years later among employees without depression at baseline. The risk of depression was related to the severity and frequency of the violent and threatening episodes. Furthermore, we found that employees exposed to work-related threats had an increased risk of getting a prescription of anti-depressive medication within a two-year period. To our knowledge, this is the first study examining the longitudinal association between exposure to work-related violence and threats and depression diagnosed by a standardized psychiatric interview and the first study on work-related violence that combines two measures of depression.

The results corroborate the findings from our recent review and meta-analyses in which we found a weighted averaged relative risk (RR) of depression, RR 1.42 (95% CI 1.31–1.54) after exposure to work-related violence and threats (8). This result was based on four studies, in which only one study, a registry-based study, explicitly addressed the risk of medically diagnosed depressive disorders; however, this was not separated from other mood disorders (9). Two of the four studies used the prescription of antidepressants as a proxy measure of depressive disorder, which we did in the second part of this study (10, 11). Madsen et al (11) used a sample of 15 246 Danish employees not using anti-depressive medication at baseline, who were linked to a national registry of prescription medication purchases to detect incidence use of antidepressants over a six years follow-up. They found that self-reported violence (threats or physical) was associated with the purchase of antidepressants, HR 1.38 (95% CI 1.09–1.75). Dement et al (10) used exposure information from workers’ compensation claims and incident reports to examine the association between violent episodes and prescriptions of antidepressants among nurses, police officers and security workers. They found a RR of 1.65 (95% CI 1.10-2.48). Neither of these studies examined violence and threats of violence separately as we did in this current study. We also found elevated HR for the associations between violence and threats of violence and the prescription of antidepressants. However, the CI for occasional and frequent violence compared to no violence were wide due to the low number of cases, but the severity-frequency index showed an association between both violence and threats of violence and the prescription of antidepressants. The study population in the study by Dement consisted of ‘high risk’ workers and might not be comparable to the more generalized working population. They reported a prevalence of antidepressants and anxiolytics of 14.8% in the reference group, which is significantly higher than the general prevalence of depression in the general population [around 6% worldwide (25)]. In the present study, participants came from a wide range of occupations, which was also the case in the study by Madsen et al (11). The baseline prevalence of prescriptions of antidepressants in the current study was 6.8%, and 4.1% in the study by Madsen et al (11).

The results are also in accordance with a newly published Danish study measuring exposure to violence by a JEM and the risk of depression measured by hospital treatment in registers (12). The modest association between being employed in jobs with a high likelihood of workplace violence and subsequent risk of diagnosed depression reported in this JEM study was depending on gender and industry, with robust association among women but not men. Our population sample mostly comprised female employees employed in jobs conducting person-related work, which is likely a high-risk occupation regarding risk of depression. This may explain the stronger association between workplace violence and depression in our study. Further, measuring work-related violence by a JEM could lead to misclassification of the exposure and a depression diagnosis based on registers only may lead to an underestimation of the effect because many cases of depression are untreated or treated entirely in primary care (26). The participation rate for the initial survey was low, 45%, which raises concerns about selection bias. Kaerlev et al (17) explored the consequences of this low response rate in an earlier study. They found that the respondents differed from non-respondents by gender, age and social class. However, using registry data also available from non-respondents they did not observe any substantial differences in sick leave and use of antidepressants according to work-unit measures of the psychosocial work environment (17). Loss to follow-up was limited, but selection may still have biased our results. We compared the characteristics of respondents and non-respondents in supplementary table A. Respondents were older and more educated than non-respondents. There were more former smokers among non-respondents, and they drank less alcohol than the respondents. Non-respondents reported more depressive symptoms than respondents did, but respondents and non-respondents did not differ regarding the SCAN depression diagnosis.

In the prescription study, we excluded employees with previous episodes of depression but in the smaller SCAN study sample we adjusted for previous depression to ensure reasonable statistical power. In supplementary analyses of the SCAN study, we excluded those with previous depression, which resulted in point estimates similar to those in the main analysis, but with wider CI as expected. The most reliable method would have been to analyze the employees with previous episodes of depression separately; unfortunately, we did not have the power to do this in the current study.

### Strengths and limitations

A major strength of the study is the combination of psychiatric interview and register data to ascertain depression. To our knowledge, this has not been done before in relation to workplace violence. Our confidence in the results is strengthened by the fact that we found an association between work-related violence and threats of violence and depression in all measures of depression when using the frequency-severity index. Regarding the exposure categories, we found the same tendency of an increased risk of depression even though the estimates of the association between violence and antidepressants had low precision.

Another important strength of our study is that in our exposure assessment we not only asked ‘have you been exposed to threats?’ and ‘have you been exposed to violence?’ like many studies do, but we asked questions in more detail, in particular about violence. We measured threats by two questions, one about verbal or written threats and one about threatening behavior, and five response categories regarding the frequency of the acts. Violence was measured by three questions with increasing severity of the violent acts and the same five response categories regarding the frequency. Furthermore, we used this information in the severity-frequency index as an attempt to measure exposure in more detail. This was not a validated tool; however, by asking about different acts we reduced the risk of differential misclassification compared to only asking one single question. The reported prevalence (37.4% for threats and 18.4% for violence) did not differ from earlier studies. The relatively large size of the final sample, and knowledge about a broad set of covariates, are further strengths of the study.

One limitation is our self-reported retrospective assessment of exposure, which could result in response bias; however, violent and threatening episodes are often severe exposures and the risk of not recalling them may be relatively small.

Another limitation is the follow-up period of 2 years. One could imagine that violence might trigger a depressive disorder without delay and that targets will recover within a few months, which has been reported in studies about traumatic life events and depression (27). In this case, an increased risk may be difficult to detect after two years. Yet we do find an increased risk in this current study. This may be because violence and threats were repeated during the two-year follow-up period, with half of the employees reporting violence and/or threats in 2007 reporting exposure in 2009 as well (data not shown). In this way threats and violence remain a long-term contextual threat. The time-lag of two years is however less important because we also used the antidepressants measure. The risk of getting antidepressants prescribed was measured throughout the two-year period by Cox regression. These results support the increased risk of depression with a higher risk of prescriptions of antidepressants in response to threats and violence although the CI were wide in relation to violence and only the severity-frequency index was statistically significant.

### Concluding remarks

We found that exposure to work-related violence or threats of violence increased the risk of ICD-10 diagnosed depression two years later and increased the risk of prescriptions of antidepressants in the same time period. Replication studies using refined and validated tools for measuring exposure to violence and threats are recommended in future research to further improve the evidence of a causal association between work-related threats and violence and depression.

## Supplementary material

Supplementary material
