# Supplementary material for: Exposure to workplace violence and threats and risk of depression: a prospective study
Source: Scand J Work Environ Health. 2021 Oct 31;47(8):582–90. doi: 10.5271/sjweh.3976 (PMC9058619; doi:10.5271/sjweh.3976)
Supplement: Supplementary material [file SJWEH-47-582-S001.pdf]

Exposure to workplace violence and threats and risk of depression: a prospective study[1]

by Laura A Rudkjoebing, MD,[2] Åse Marie Hansen, PhD, Reiner Rugulies, PhD, Henrik Kolstad, PhD, Jens Peter Bonde, PhD

1. *Supplementary Material*
2. *Correspondence to: Laura Aviaja Rudkjoebing, Department of Occupational and Environmental Medicine, Bispebjerg University Hospital, Bispebjerg Bakke 23, DK-2400 Copenhagen NV, Denmark. [E-mail: Laura.aviaja.rudkjoebing.01@regionh.dk]*

Table A. Baseline characteristics (2007) of respondents and non-respondents in 2009. Odds ratios (OR) and 95 % confidence intervals (CI). OR measuring the probability of respond to the questionnaire in 2009 for each category given the specified reference.

	**Non-respondents 2009**		**Respondents 2009**			
	N total 1280	%	N total 3209	%	OR	95% CI
**Gender**						
Female	1002	78	2519	79	1	
Male	278	22	690	21	0.99	0.84-1.16
**Age groups**						
19-29	152	12	252	8	1	
30-39	363	28	726	23	1.21	0.95-1.53
40-49	362	28	972	30	1.62	1.28-2.05
50-59	354	28	1103	34	1.88	1.49-2.38
>59	49	4	156	5	1.92	1.32-2.81
**Higher education**						
<3 years	328	26	563	18	1	
3-4 years	831	65	2247	70	1.58	1.35-1.85
>4 years	116	9	389	12	1.95	1.53-2.50
**Smoking**						
Never smoked	554	44	1526	48	1	
Former Smoker	412	32	1159	36	1.02	0.88-1.19
Smoker	328	24	502	16	0.59	0.50-0.70
**Alcohol**						
≤7 units/week	1002	79	2408	76	1	
>7 units/week	259	21	769	24	1.24	1.05-1.45
**Traumatic life events**						
No	797	62	2066	64	1	
Yes	483	38	1143	36	0.91	0.80-1.04
**Family depression**						
No	911	73	2291	73	1	
Yes	344	27	863	27	1.00	0.86-1.16
**Earlier depression**						
No	1039	84	2696	86	1	
Yes	199	16	428	14	0.83	0.69-1.00
**Depressive symptoms**						
No	1163	92	2996	94	1	
Yes	107	8	203	6	0.74	0.58-0.94
**Depression, ICD-10**						
No	1250	98	3139	98	1	
Yes	30	2	70	2	0.93	0.60-1.43
**Neurotic**						
No	1049	82	2702	84	1	
Yes	231	18	507	16	0.85	0.72-1.01
**Threats**						

Never	763	60	2030	64	1	
Occasionally	399	31	917	29	0.86	0.75-1.00
Frequently	111	9	241	7	0.82	0.64-1.04
**Violence**						
Never	1013	80	2620	82	1	
Occasionally	184	14	391	12	0.82	0.68-0.99
Frequently	71	6	172	6	0.94	0.70-1.25

Table B. Risk of newly-onset depression from 2007 to 2009 and from 2009 to 2011 by self-reported threats of violence and violence in 2007 and 2009, respectively. With exclusion of employees with previous episodes of depression. Odds ratios (OR) and 95 % confidence intervals (CI).

	Number of observations	New cases of depression SCAN	%	OR_{crude}	95% CI	OR_{adj}	95% CI
**Threats:**							
Never	3511	44	1.3	1		1	
Occasionally	1380	14	1.0	0.81	0.44-1.48	0.71	0.37-1.34
Frequently	346	9	2.6	2.10	1.02-4.34	2.02	0.92-4.43
Continuous*				1.23	0.82-1.84	1.17	0.76-1.82
Severity-frequency index**				1.07	0.99-1.16	1.06	0.97-1.15
**Violence:**							
Never	4317	49	1.1	1		1	
Occasionally	606	12	2.0	1.78	0.92-3.47	1.41	0.67-2.98
Frequently	253	5	2.0	1.95	0.77-4.94	1.66	0.61-4.51
Continuous*				1.49	1.02-2.18	1.32	0.84-2.07
Severity-frequency index**				1.12	1.02-1.23	1.11	0.97-1.27

adj adjusted for age, gender, depressive symptoms, family history of depression, higher education, alcohol, smoking, traumatic life events and neuroticism.

*Increase in OR by 1 on a scale ranging from 0-3.

** Increase in OR by 1 on the severity-frequency scale (ranging from 0-12 for threats and 0-24 for violence).

Figure A. The distribution of the severity frequency index for threats.

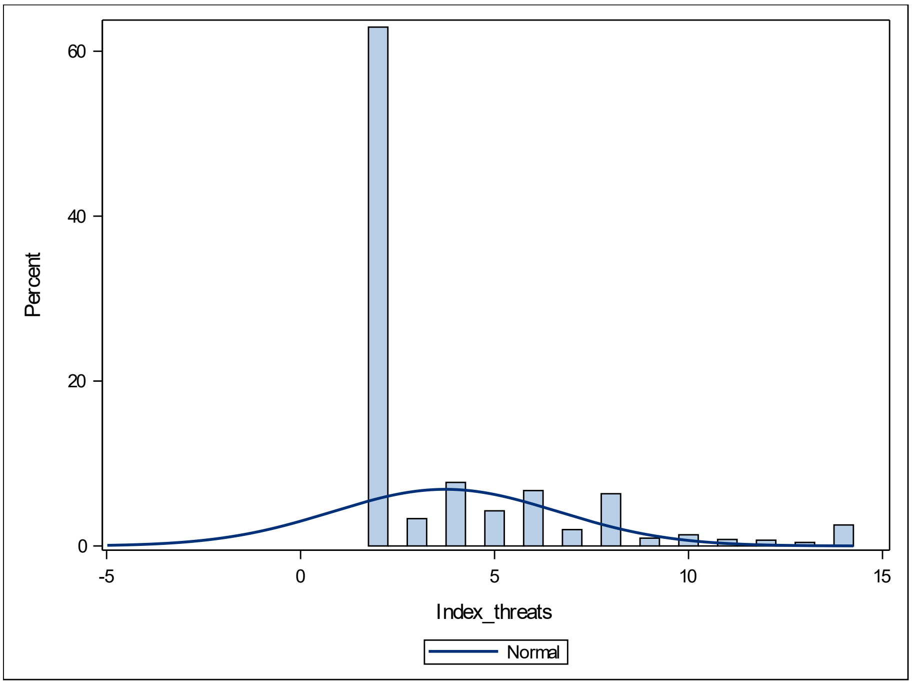

Figure B. The distribution of the severity frequency index for violence.

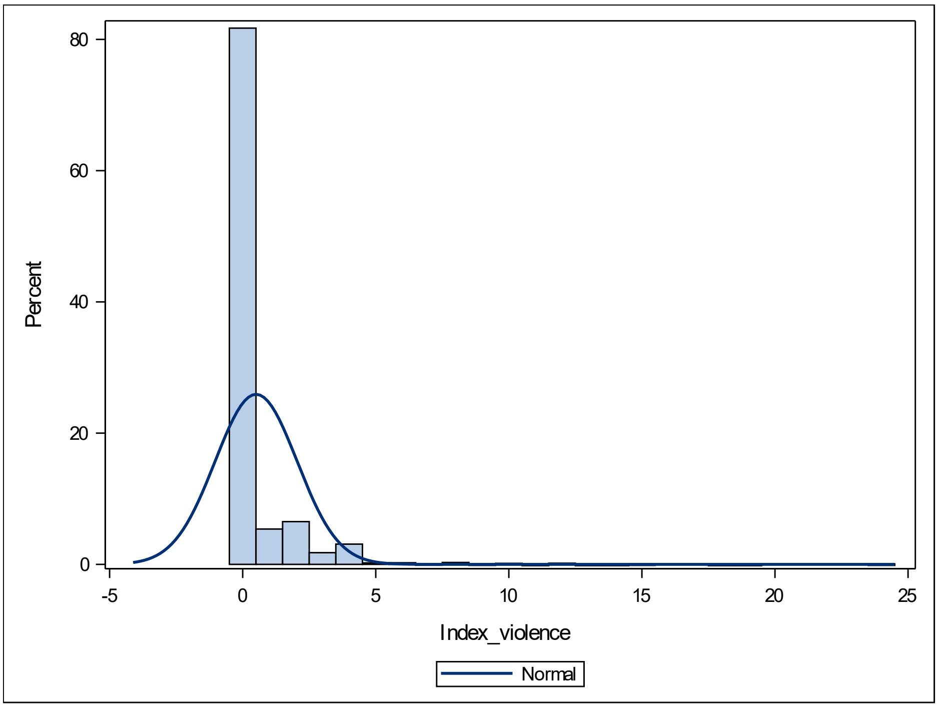
Percent
80
60
40
20
0
-5
0
5
10
15
20
25
Index_violence
Normal
